# Sex differences in hippocampal β-amyloid accumulation in the triple-transgenic mouse model of Alzheimer’s disease and the potential role of local estrogens

**DOI:** 10.3389/fnins.2023.1117584

**Published:** 2023-03-08

**Authors:** Yu-Ting Hu, Xin-Lu Chen, Ya-Nan Zhang, Hugo McGurran, Jochem Stormmesand, Nicole Breeuwsma, Arja Sluiter, Juan Zhao, Dick Swaab, Ai-Min Bao

**Affiliations:** ^1^Affiliated Mental Health Center and Hangzhou Seventh People’s Hospital, Zhejiang University School of Medicine, Hangzhou, China; ^2^Department of Neurobiology and Neurology of the Second Affiliated Hospital, Zhejiang University School of Medicine, Hangzhou, China; ^3^NHC and CAMS Key Laboratory of Medical Neurobiology, MOE Frontier Science Center for Brain Research and Brain-Machine Integration, School of Brain Science and Brain Medicine, Zhejiang University, Hangzhou, China; ^4^Netherlands Institute for Neuroscience, The Royal Netherlands Academy of Arts and Sciences, Amsterdam, Netherlands

**Keywords:** Alzheimer’s disease, triple-transgenic AD mouse, β-amyloid, 17β-estradiol, sex differences

## Abstract

**Introduction:**

Epidemiological studies show that women have a higher prevalence of Alzheimer’s disease (AD) than men. Peripheral estrogen reduction during aging in women is proposed to play a key role in this sex-associated prevalence, however, the underlying mechanism remains elusive. We previously found that transcription factor early growth response-1 (EGR1) significantly regulates cholinergic function. EGR1 stimulates acetylcholinesterase (AChE) gene expression and is involved in AD pathogenesis. We aimed to investigate whether the triple-transgenic AD (3xTg-AD) mice harboring PS1^*M*146*V*^, APP*^Swe^*, and Tau^*P*301*L*^ show sex differences in β-amyloid (Aβ) and hyperphosphorylated tau (p-Tau), the two primary AD hallmarks, and how local 17β-estradiol (E2) may regulate the expression of EGR1 and AChE.

**Methods:**

We first sacrificed male and female 3xTg-AD mice at 3–4, 7–8, and 11–12 months and measured the levels of Aβ, p-Tau, EGR1, and AChE in the hippocampal complex. Second, we infected SH-SY5Y cells with lentivirus containing the amyloid precursor protein construct C99, cultured with or without E2 administration we measured the levels of extracellular Aβ and intracellular EGR1 and AChE.

**Results:**

Female 3xTg-AD mice had higher levels of Aβ compared to males, while no p-Tau was found in either group. In SH-SY5Y cells infected with lentivirus containing the amyloid precursor protein construct C99, we observed significantly increased extracellular Aβ and decreased expression of intracellular EGR1 and AChE. By adding E2 to the culture medium, extracellular Aβ(1–42) was significantly decreased while intracellular EGR1 and AChE expression were elevated.

**Discussion:**

This data shows that the 3xTg-AD mouse model can be useful for studying the human sex differences of AD, but only in regards to Aβ. Furthermore, in vitro data shows local E2 may be protective for EGR1 and cholinergic functions in AD while suppressing soluble Aβ(1–42) levels. Altogether, this study provides further in vivo and in vitro data supporting the human epidemiological data indicating a higher prevalence of AD in women is related to changes in brain estrogen levels.

## 1. Introduction

Alzheimer’s Disease (AD) is more prevalent in women than in men even after correcting for the longer lifespan of women and also exhibit more severe cognitive decline during the course of AD (Viña and Lloret, 2010; Laws et al., 2016). It has been proposed that the significant reduction of peripheral estrogen levels during aging in women plays a key role for this sex difference in AD (Henderson, 2006; Sahab-Negah et al., 2020). To support this, 17β-estradiol (E2) was found to be neuroprotective (Dye et al., 2012), was reported to reduce the production of β-amyloid (Aβ) (Chang et al., 1997; Xu et al., 1998), and stimulate its degradation (Liang et al., 2010). In addition, E2 prevents the formation of hyperphosphorylated Tau (p-Tau) *in vitro* (Alvarez-de-la-Rosa et al., 2005). Both Aβ and p-Tau are the neuropathological hallmarks of AD (Braak and Braak, 1991), though their biological relationship to each other, and the influence of sex hormones on them are still poorly understood.

β-amyloid is found in the brain in two main forms, Aβ(1–40) and Aβ(1–42), which contain 40 and 42 amino acids, respectively. Aβ(1–40) is significantly more abundant in the brain, however, Aβ(1–42) has been shown in many studies to be the primary driver of pathological seeding of soluble aggregates (e.g., oligomers, protofibrils) and insoluble amyloid fibrils (Lambert et al., 1998; Wang et al., 2000; Bode et al., 2017). In human neuropathological studies of AD patients, Aβ is typically found as insoluble extracellular plaques, however, this is often a late stage of Aβ aggregation, and researchers have revealed that soluble Aβ oligomers, which often precedes the plaques in AD, drive the impairment in synaptic plasticity and cognitive function in AD (Walsh et al., 2002; Lacor et al., 2007; Shankar et al., 2008). As such, soluble Aβ oligomers may be a causative agent in the development and progression of AD (Hayden and Teplow, 2013; Tolar et al., 2021). Recent clinical trials support this idea, as only agents targeting soluble Aβ oligomers show clinical efficacy, while agents targeting amyloid monomers or plaques failed to show clinical effects in AD patients (for review see Tolar et al., 2021).

Brain cholinergic activity is crucial for cognition and is affected in the AD process (Francis et al., 1999; Hampel et al., 2018). This hypothesis has been proposed due to findings showing depleted presynaptic cholinergic markers in the cerebral cortex of AD patients, which may in turn begin or accelerate processes of cognitive decline (Davies and Maloney, 1976). In addition, in late AD stages there is severe neurodegeneration of the nucleus basalis of Meynert (NBM), which is the primary source of cortical cholinergic innervation (Whitehouse et al., 1981), and correspondingly, clinical data indicates cholinergic antagonists and agonists can impair and enhance memory, respectively (Salehi et al., 1994; Mesulam, 2013). Moreover, by preventing the degradation of acetylcholine using cholinesterase inhibitors were shown to significantly improve symptoms in patients with AD (Lleó et al., 2006). Adding to this cholinergic hypothesis of AD at a biological level, we and other groups have found that the early growth response-1 (EGR1), a zinc finger transcription factor, plays a significant role in regulating cholinergic functions, such as upregulating gene expression of acetylcholinesterase (AChE) (Hu et al., 2019) and of choline acetyltransferase (Quirin-Stricker et al., 1997). EGR1 is also known to promote neuronal plasticity and memory formation (Knapska and Kaczmarek, 2004; Veyrac et al., 2014). We have found that EGR1 remains stable, or slightly increased, in the NBM and the prefrontal cortex (PFC) in early AD stages, possibly contributing to stimulating neuronal activity in early AD and thereby preventing early symptomatology (Zhu et al., 2016; Hu et al., 2019). However, EGR1 was also found to promote Aβ production by upregulating the PSEN2 gene in neurons, which is a crucial cofactor in γ-cleavage of amyloid precursor protein (APP) (Renbaum et al., 2003; Qin et al., 2016, 2017). Moreover, Aβ(1–42) was found to bind with high affinity and block presynaptic α7nAChR, an ionotropic acetylcholine receptor with high permeability to calcium, thus inhibiting acetylcholine release (Wang et al., 2000).

In order to study the sex differences in accumulation of Aβ and p-Tau and the potential impact on the expression of EGR1 and AChE, we utilized the triple-transgenic mouse model of AD (3xTg-AD). The 3xTg-AD mouse is one of the main transgenic models of AD, and one of the few genetically overexpressing both p-Tau and Aβ. The model contains three mutations associated with familial AD: PS1^*M*146*V*^, APP*^Swe^*, and Tau^*P*301*L*^ (Oddo et al., 2003). However, whether this AD mouse model shows sex differences in the accumulation of Aβ or p-Tau at all remains elusive, and may depend on e.g., the age of the mice (Hirata-Fukae et al., 2008; Carroll et al., 2010a; Belfiore et al., 2019). To that end, we examined the hippocampal complex of 3xTg-AD mice throughout the early stages of the disease, i.e., from 3 months old to 12 months old. In this study we found that female 3xTg-AD mice had higher Aβ levels than males throughout this age range, while no p-Tau present in these mice. EGR1 and AChE levels did not vary significantly between 3xTg-AD and WT mice or between sexes, or among the age groups. While EGR1 and AChE levels were both significantly negatively correlated with the contents of soluble Aβ(1–42) in pooled 3xTg-AD males but not females. We then explored *in vitro* whether E2 may affect the expression of Aβ, as well as the expression of EGR1 and AChE. We found that increased extracellular Aβ may decrease the expression of intracellular EGR1 and AChE, and E2 reduces extracellular Aβ(1–42), leading to increased expression of intracellular EGR1 and AChE. Altogether, this data indicates the 3xTg-AD model is useful for studying the sex difference of Aβ in early AD, and that local E2 may exert a protective effect in AD brains by activating EGR1 accompanied with reduced soluble Aβ(1–42), and thereby maintaining synaptic and cognitive functions in early AD.

## 2. Materials and methods

### 2.1. 3xTg-AD mice study

#### 2.1.1. Animal and tissue preparation

Two pairs of 3xTg-AD parent mice obtained from Jackson Laboratory (cat no. 34,830, Bar Harbor, ME, USA) were maintained under a 12/12 h light/dark cycle at 25°C and provided with food and water *ad libitum*. Offspring were genotyped to confirm the expression of PS1^*M*146*V*^, APP*^Swe^*, and Tau^*P*301*L*^ and were randomly placed into three different age groups, i.e., to be sacrificed at 3–4, 7–8, and 11–12 months, for subsequent studies. Mice were sacrificed by carbon dioxide followed by cervical dislocation. Animal handling and experimental procedures were carried out in accordance with the Animal Experimental Committee of the Royal Netherlands Academy of Arts and Sciences.

The brains of 18 3xTg-AD mice (three male, three female mice per age group) were fixed by perfusion with 4% paraformaldehyde (w/v) in phosphate-buffered saline. Next, the brains were soaked in 20% sucrose (w/v) at 4°C until they sank to the bottom. The brains were frozen, serially sectioned in the coronal plane at 20 μm thickness on a cryostat (Leica, CM1850 UV, Nussloch, Germany). The start of the coronal plane was at about 3.0 mm posterior to the bregma where the lateral ventricle terminated. Sections were stored at –80°C until used for immunohistochemistry. Six wild type (WT) mice were used as negative controls for their sex- and age-matched group.

An additional 50 3xTg-AD mice (23 males, 27 females) were obtained. Each age group contained 7–9 male and 8–10 female mice. The brains were rapidly removed and put on ice. The hippocampal complex from the right hemisphere was isolated for ELISA, and the left-side hippocampal complex was isolated for mRNA analysis. The mRNA levels of EGR1 and AChE were also detected in 52 WT mice (9–10 males and 7–9 females per age group).

#### 2.1.2. Nissl (thionine) staining

Thionine staining was performed to visualize the anatomical structure. All sections were first air-dried at room temperature (RT) for 15 min, after which they were washed 3 × 10 min in Tris-buffered saline (TBS) and stained in thionine solution for 5 min. Sections were then washed in running tap water until the water was clear of thionine. Sections were dehydrated in graded ethanols (50 to 100%, 5 min each), cleared in xylene for 2 × 10 min, cover slipped with Entellan and dried in a fume hood overnight.

#### 2.1.3. Immunohistochemistry for Aβ and p-Tau

Frozen sections were first air-dried and washed in TBS as above. For Aβ staining, sections were then briefly washed in antigen retrieval buffer (0.01 M citrate buffer + 0.05% tween-20, pH 6.0), preheated for approximately 5 min until boiling, then 2 × 5 min at 800 W in a microwave. After cooling to RT, sections were washed in aqua dest, after which they were subjected to 10 min 70% fresh formic acid (FA) treatment. Sections were then washed 3 × 10 min in TBS and pre-incubated in 5% milk powder (ELK, Amsterdam, Netherlands) TBS (w/v) for 30 min at RT to reduce the background. Primary antibody incubation was performed with monoclonal mouse anti-Aβ 4G8 (Signet, Norwell, MA, USA; 1:20,000) in super-mix [0.05 M Tris-HCl + 0.15 M NaCl + 0.25% gelatin + 0.05% Triton X-100 (v/v), pH 7.6] with 5% milk powder overnight at RT in a moist chamber. For p-Tau staining, after deparaffinization and hydration, sections were directly washed 3 × 10 min in TBS and pre-incubated as above. Primary antibody, the mouse monoclonal anti-p-Tau AT8 (Thermo, Waltham, MA, USA; 1:300), was incubated in super-mix with 5% milk powder for 1 h at RT and overnight at 4°C in a moist chamber.

On the second day, all sections were washed 3 × 10 min in TBS and then incubated with horse anti-mouse-HRP secondary antibody (DAKO, Glostrup, Denmark; 1:400) in super-mix for 1 h at RT. Sections were then developed with DAB substrate solution (0.5 mg/ml DAB + 0.2% nickel ammonium sulfate + 0.01% H_2_O_2_ in TBS) for 18 min. The reaction was stopped in aqua dest. It should be noted that we did not find p-Tau positive signals.

#### 2.1.4. Image analysis for quantitative immunohistochemistry

**FIGURE 1:**
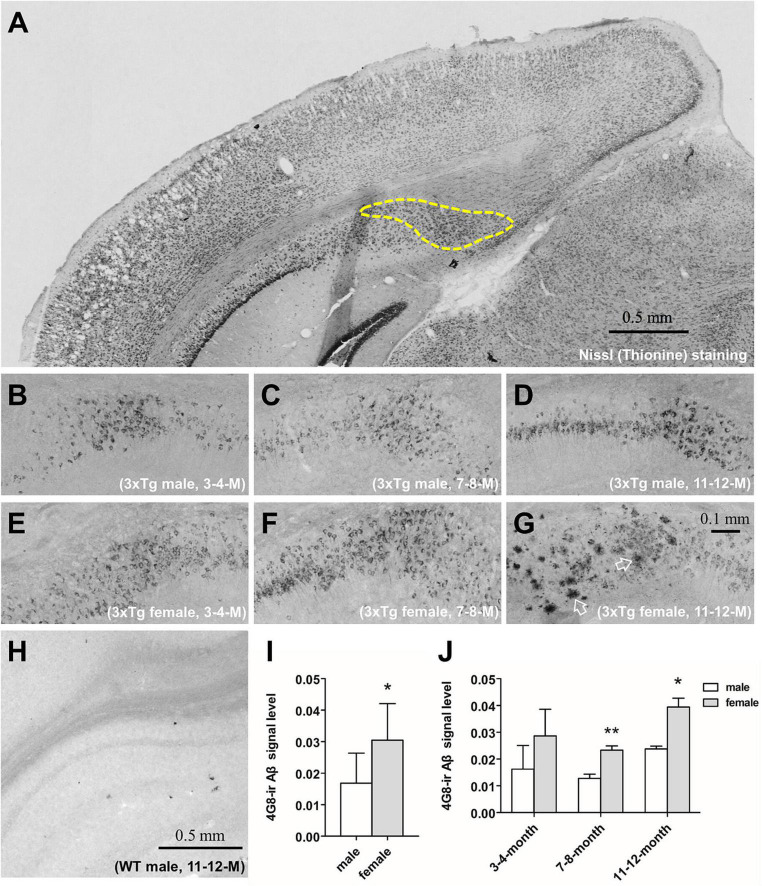
The immunohistochemical analysis of the β-amyloid (Aβ) and signal quantification in the subiculum of 3xTg-AD mice. Representative coronal section of the brain of 11–12 months-old male 3xTg-AD mouse with thionine-stained subiculum outlined by a dashed line **(A)**. 4G8-stained Aβ in the subiculum of male **(B–D)** and female **(E–G)** 3xTg-AD mice of 3–4 (left), 7–8 (middle) and 11–12 (right) months old. Open arrows show amyloid plaques. 4G8 staining in a 11–12 months male wild type mouse **(H)**. Aβ signal quantification in the pooled male (*n* = 9) and female (*n* = 9) mice **(I)**, as well as in each age group (*n* = 3 per group) **(J)**. Data are presented as mean ± standard deviation. (*) compared with corresponding males. *P ≤ 0.05, **P ≤ 0.01.

Images from brain sections were taken by a black and white camera (SONY XC-77E) mounted on a microscope (Zeis Axioskop with Plan-NEOFLUAR Zeiss objectives, Carl Zeiss GmbH, Oberkochen, Germany) at 10 × magnification. The border of the subiculum was defined according to the Mouse Brain in Stereotaxic Coordinates (Paxinos and Franklin, 2001) and was showed in Figure 1A. Images were analyzed using Image Pro 6.3 software, and signal quantification was based on optical density (OD) measurements and thresholding, as described in detail before (Zhu et al., 2016). In brief: The threshold was set at two times the OD value of the individual background. Within the outlined area, the computer then determined the OD values (density mask) and the surface area covered by immunocytochemical signal (area mask). The integrated optical density (IOD) was calculated by multiplying density mask with area mask. Since the size of the subiculum varied across mice, the final value was corrected by dividing the IOD value by the total outlined area to obtain the corrected IOD (cIOD) value. The values of both hemispheres’ subiculum were analyzed together. Researchers were blind to the identity of all the sections.

#### 2.1.5. ELISA for Aβ

Sample preparation was performed according to the manufacturer’s instruction (BioLegend, San Diego, CA, USA). Briefly, frozen hippocampal complexes were mechanically homogenized in ice-cold TBS supplemented with complete protease inhibitor cocktail tablets (Roche, Munich, Germany), with one tablet in 50 ml TBS, at 10 ml per 1 g tissue. Homogenates were then spun down at 350,000 g for 20 min at 4°C by ultracentrifugation (TLA-120.1 fixed-angle rotor, Beckman Coulter, Brea, CA, USA). The resultant supernatant was collected, representing the soluble Aβ fraction (TBS-Aβ). The pellet was subsequently dissolved in 70% FA and centrifuged again as described above. The resultant supernatant was collected, representing the insoluble Aβ fraction (FA-Aβ). Before the ELISA assay, the bulk of FA buffer in FA-Aβ extracts was evaporated off down to 10% of the original volume using a nitrogen stream. FA extracts were then neutralized to around pH 7.0 with 1 M NaOH. Aβ(1–40) and Aβ(1–42) levels were measured separately by ELISA according to the manufacturer’s instructions (842,301 and 842,401, BioLegend, San Diego, CA, USA). Optical density (OD) values were read at 620 nm by Varioskan Flash microplate reader (Thermo, Waltham, MA, USA).

#### 2.1.6. Quantitative polymerase chain reaction (qPCR) for EGR1 and AChE

The hippocampal complex was homogenized in 1 ml ice-cold TRIsure (38,032, Bioline, Luckenwalde, Germany) by using ultra-turrax at RT. 0.2 ml chloroform was added and well-mixed, the samples were then stored at RT for 5 min, after which they were spined down at 12,000 g for 15 min at 4°C. The aqueous phase was obtained and equal volume of ice-cold isopropanol was added followed by 1 μl GenElute-LPA (56,575, Sigma, St. Louis, MO, USA). Samples were then precipitated overnight at –80°C. The next day the samples were spined down at 12,000 g for 30 min at 4°C. Pellets were washed in 0.5 ml 75% ethanol after removing the supernatant. After the pellets were suspended in 20 μl filtered MilliQ-water and fully dissolved, the RNA samples were diluted 1:10 and the concentrations were measured by NanoDrop ND-1000 spectrophotometer (Thermo, Waltham, MA, USA).

cDNA was synthesized using the QuantiTect reverse transcription kit (205,313, QIAGEN, Hilden, Germany), cDNA was diluted to 1:10 for qPCR by 7,300 Real Time PCR System (Thermo, Waltham, MA, USA). Geomean value of two housekeeping genes, glyceraldehyde phosphate dehydrogenase (GAPDH) and hypoxanthine guanine phosphoribosyl transferase (HPRT), were calculated. Primers for EGR1 and AChE were same as our previous study (Hu et al., 2019).

### 2.2. Cell line study

#### 2.2.1. Cell culture and lentivirus infection

SH-SY5Y cells (ATCC CRL-2266) were cultured in mediumX containing Dulbecco’s Modified Eagle Medium/Nutrient Mixture F-12 (DMEM/F-12; 10,565,018, Thermo, Waltham, MA, USA) supplemented with 10% fetal bovine serum (FBS; 16000044, Thermo, Waltham, MA, USA) together with 1% penicillin and streptomycin (15,070,063, Thermo, Waltham, MA, USA), and were incubated at 37°C in a humidified chamber with 5% CO_2_.

Lentivirus containing the construct of C-terminal 99 amino acids of APP (Ubi-C99-3FLAG-SV40-EGFP-IRES-puromycin, LV-C99), and its control lentivirus (LV-CTR) were produced by Genechem (Shanghai, China). Lentivirus infection was performed according to the manufacturer’s instructions. In brief: SH-SY5Y cells were seeded at 10^5^ cells per well and cultured in 6-well plate for 24 h prior to infection. C99-SH-SY5Y cells were obtained by adding 1 ml LV-C99 solution (5 × 10^6^ TU LV-C99 + 50 μg polybrene in mediumX) to each well and incubating for 5 min at RT before putting the plate back to the incubator. The corresponding control cells (CTR-SH-SY5Y) were obtained by LV-CTR infection following the same procedure. Infection ratio was determined after 48 h by NovoCyte flow cytometer (Agilent, Santa Clara, CA, USA).

#### 2.2.2. E2 administration

Prepare mediumY: DMEM/F-12 HEPES without phenol red (11,039,021, Thermo, Waltham, MA, USA) supplemented with 10% FBS without E2 (04-201-1B, Biological Industries, Cromwell, CT, USA) together with 1% penicillin and streptomycin. The original mediumX in the 6-well plate was substituted by mediumY 24 h after seeding. E2 stock solution (1 mM) was prepared by dissolving E2 powder (E2758, Sigma, St. Louis, MO, USA) in 10% ethanol in DMEM/F-12 HEPES, and was added into each well to a final concentration of 1 μM E2, and incubated for another 24 h before performing all the measurements.

#### 2.2.3. ELISA for extracellular Aβ

The medium was collected and spun down at 12,400 g for 1 min at 4°C by ultracentrifugation before the resultant supernatant was collected. Extracellular Aβ(1–40) and Aβ(1–42) levels were measured separately according to the manufacturer’s instructions (KHB3411 and KHB3544, Thermo, Waltham, MA, USA). OD values were read at 450 nm by Varioskan Flash microplate reader.

#### 2.2.4. qPCR and western blot for EGR1 and AChE

For qPCR measurement, 1 ml ice-cold Trizol was added per well and the cell lysate was collected. The rest steps were the same as shown in Part 1. HPRT was used as housekeeping gene. Primers for EGR1 and AChE were the same as in our previous study (Hu et al., 2019). The western blot was adapted from the protocol as described before (Hu et al., 2019).

### 2.3. Statistical analysis

For 3xTg-AD mice study, Student’s *t*-test was applied for comparing Aβ, EGR1 and AChE levels between male and female mice, either in 3xTg-AD or in WT mice. Two-way ANOVA was applied for comparing EGR1 and AChE levels among age groups between 3xTg-AD and WT mice. Correlations between molecules were evaluated with the Pearson test. For the cell line study, Student’s *t*-test was applied for comparing Aβ, EGR1 and AChE levels between C99-SH-SY5Y and its CTR-SH-SY5Y cells, and between C99-SH-SY5Y cells cultured with E2 and without E2. Data was represented as mean ± standard deviation. Statistics were performed with SPSS 17.0. *P* ≤ 0.05 was considered to be significant.

## 3. Results

### 3.1. 3xTg-AD mice study

#### 3.1.1. Female 3xTg-AD mice presented higher Aβ loads and faster Aβ accumulation in the hippocampal complex

β-amyloid was found using immunohistochemistry in the cytoplasm of hippocampal neurons in 3xTg-AD mice at 3–4, 7–8, and 11–12 months of age in both males (Figures 1B–D) and females (Figures 1E–G). Only at 11–12 months of age, cored Aβ plaques appeared in the subiculum of the hippocampus of female (Figure 1G) but not male 3xTg-AD mice (Figure 1D). Aβ signal in the subiculum was stronger in female mice overall (*P* = 0.02, Figure 1I), and especially in the 7–8 months (*P* = 0.009) and the 11–12 months (*P* = 0.04, Figure 1J) age groups. Aβ signal was absent in all WT mice (Figure 1H).

**FIGURE 2:**
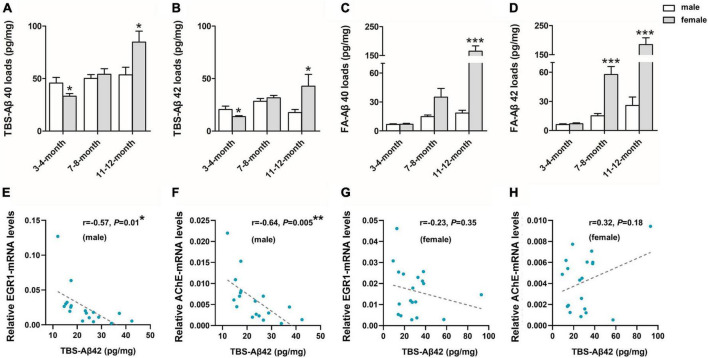
β-amyloid (Aβ) expression in the hippocampal complex of 3xTg-AD mice and the correlation with early growth response-1 (EGR1) and acetylcholinesterase (AChE) levels. Expression of soluble Tris-buffered saline (TBS)-Aβ(1–40) **(A)**, –Aβ(1–42) **(B)**, and insoluble FA-Aβ(1–40) **(C)**, –Aβ(1–42) **(D)** in the hippocampal complex of both male and female 3xTg-AD mice of different age groups. In each respective age group (3–4, 7–8, 11–12 months) sample numbers are; for TBS-Aβ, *n* = 7, 8, 7 (male) and *n* = 8, 8, 6 (female); for FA-Aβ, *n* = 6, 6, 6 (male) and *n* = 7, 6, 5 (female). The correlations between TBS-Aβ(1–42) load with the mRNA level of EGR1 **(E,G)** and AChE **(F,H)** in pooled males (*n* = 18) and females (*n* = 18), respectively. Aβ loads were measured by ELISA and presented as picogram of Aβ per milligram of the hippocampal complex tissue (pg/mg). mRNA level was measured by qPCR. Data are presented as mean ± standard deviation. (*) compared to corresponding males. *P ≤ 0.05, **P ≤ 0.01, ***P ≤ 0.001.

To further investigate the sex difference of Aβ pathology, we performed an ELISA for both soluble Aβ(1–40/42) fractions [TBS-Aβ(1–40), TBS-Aβ(1–42)] fractions as well as the insoluble Aβ(1–40/42) fractions [FA-Aβ(1–40), FA-Aβ(1–42)], since soluble Aβ oligomers may be a causative agent in the development of AD. TBS-Aβ(1–40) (Figure 2A) and TBS-Aβ(1–42) (Figure 2B) levels were both significantly lower in females than in males at 3–4 months (*P* < 0.05, *P* < 0.05) while in 11–12 months-old mice, females showed significantly higher TBS-Aβ(1–40) and TBS-Aβ(1–42) levels compared to males (*P* = 0.03, *P* = 0.04). FA-Aβ(1–40) (Figure 2C) and FA-Aβ(1–42) (Figure 2D) levels were significantly higher in females of both 7–8 (*P* = 0.05 and *P* < 0.001) and 11–12 months (*P* < 0.001 and *P* < 0.001) compared to their age-matched males.

While it should be noted that we did not find p-Tau signals in the brains of 3xTg-AD mice until the age of 11–12 months.

#### 3.1.2. EGR1 and AChE levels were stable in the hippocampal complex of 3xTg-AD and WT mice

There was no significant change in the level of EGR1 [*F*(2, 96) = 0.09, *P* = 0.92] or AChE [*F*(2, 96) = 0.32, *P* = 0.72] between 3xTg-AD and WT mice across the three age groups. Neither the mouse type or the age has a significant effect on the expression of EGR1 (*P* ≥ 0.54) and AChE (*P* ≥ 0.35). No sex difference in EGR1 and AChE mRNA levels was observed either in 3xTg-AD (*P* ≥ 0.10, *P* ≥ 0.13) or WT (*P* ≥ 0.83, *P* ≥ 0.38) mice (Figures not shown).

In 3xTg-AD mice, EGR1 and AChE mRNA levels were significantly positively correlated for the entire group and within each age group or sex, while this correlation only showed a trend in females of 11–12 months-old. EGR1 and AChE were also positively correlated in both the entire group and when separated by age or sex subgroups in WT mice, with the exception of females at 7–8 months of age see details in Supplementary Table 1.

In addition, EGR1 and AChE mRNA were both significantly negatively correlated with the contents of soluble Aβ(1–42) (*r* = –0.57, *P* = 0.01, Figure 2E and *r* = –0.64, *P* = 0.005, Figure 2F, respectively) in pooled 3xTg-AD males but not females (*r* = –0.23, *P* = 0.35, Figure 2G and *r* = 0.32, *P* = 0.18, Figure 2H, respectively).

### 3.2. Cell line study

#### 3.2.1. Increased extracellular Aβ is accompanied by decreased intracellular EGR1 and AChE

**FIGURE 3:**
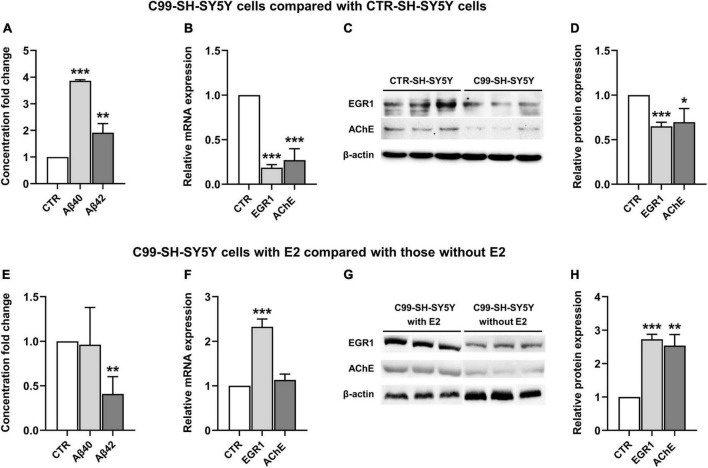
The effect of 17β-estradiol (E2) on the expression of early growth response-1 (EGR1) and acetylcholinesterase (AChE) and concentration of Aβ(1–40) and Aβ(1–42) in C99-SH-SY5Y cells. Concentration fold change of Aβ(1–40) and (1–42) **(A)**, relative mRNA level of EGR1 and AChE **(B)** and their representative western blot results **(C,D)** in C99-SH-SY5Y cells compared with SH-SY5Y cells. Concentration fold change of Aβ(1–40) and (1–42) **(E)**, relative mRNA level of EGR1 and AChE **(F)** and their representative western blot results **(G,H)** in C99-SH-SY5Y cells with E2 added to the medium compared those without E2 added to the medium. β-amyloid (Aβ) loads were measured by ELISA. Data are presented as mean ± standard deviation. (*) compared to corresponding control cells. *P ≤ 0.05, **P ≤ 0.01, ***P ≤ 0.001.

In comparison with the CTR-SH-SY5Y cells, Aβ(1–40) and Aβ(1–42) were significantly increased in C99-SH-SY5Y cells medium (*P* < 0.001 and *P* = 0.01, respectively, Figure 3A), while EGR1 and AChE were significantly decreased in both mRNA (*P* < 0.001 and *P* < 0.001, Figure 3B) and protein (*P* < 0.001 and *P* = 0.03, Figures 3C, D) levels in C99-SH-SY5Y cells.

#### 3.2.2. E2 inhibited extracellular Aβ(1–42) and increased intracellular EGR1 and AChE

Adding E2 to the medium of C99-SH-SY5Y cells reduced Aβ(1–42) but not Aβ(1–40) (*P* = 0.006 and *P* = 0.88, respectively, Figure 3E) compared to C99-SH-SY5Y cells without supplemented E2. Moreover, we observed elevated mRNA (*P* < 0.001, Figure 3F) and protein (*P* < 0.001, Figures 3G, H) levels of EGR1, as well as increased AChE protein level (*P* = 0.001, Figures 3G, H) despite its unchanged mRNA level (*P* = 0.15, Figure 3F) in E2-supplemented C99-SH-SY5Y.

## 4. Discussion

We observed female 3xTg-AD mice had higher levels and faster accumulation of Aβ, but without p-Tau accumulation, in the hippocampal complex from 3 to 12 months of age compared to males. In addition, the subiculum was the major structure of hippocampal complex that first showed amyloid plaques in females but not males at the age of 11–12 months. In C99-SH-SY5Y cells overexpressing Aβ, we observed decreased EGR1 and AChE levels. Adding E2 to the medium significantly decreased extracellular Aβ(1–42), which was accompanied by increased intracellular expressions of EGR1 and AChE. Altogether, our findings indicate that the 3xTg-AD mice is an appropriate model to study the mechanism of the sex difference in early Aβ, but not p-Tau, expression in AD, and that, *in vitro*, local E2 may regulate the interaction among Aβ(1–42), EGR1 and cholinergic functions.

Studies have previously found that female 3xTg-AD mice had a higher plaque burden in the subiculum than males at approximately 13–14 months (Hirata-Fukae et al., 2008; Carroll et al., 2010a), however, our findings brought such a sex difference back to as early as at 11–12 months old. It should be noted that unlike Clinton et al. (2007), who reported there were no sex differences in the levels of soluble and insoluble Aβ(1–40) and Aβ(1–42) in the whole brain of 3xTg-AD mice from 2 to 15 months of age, we found higher soluble Aβ(1–40) and Aβ(1–42) expressions in the hippocampal complex of female than in male mice by 11–12 months old. This study, however, did also note that the level of soluble Aβ(1–40) was slightly higher, though not significant, in 2 and 6 months old males; and soluble Aβ(1–42) level was also slightly higher, but not significant, in 6 months-old males compared with females (Clinton et al., 2007). This may then support the higher soluble Aβ levels we observed in the 3–4 months-old male mice compared to their age-matched females. Furthermore, we found more insoluble Aβ(1–40) and Aβ(1–42) expressions from the age of 7–8 months in females. This indicates that the accumulation of Aβ is area-dependent and that sex differences in AD may be local but not general, with the subiculum being one of the regions earlier and more severely affected by Aβ in females.

Although 3xTg-AD female mice have progressive Aβ accumulation from 3 to 4 months of age, their peripheral E2 levels do not show significant change compared with age-matched WT mice (Clinton et al., 2007; Zhao et al., 2011). Previous studies also found that ovariectomy (OVX) in 3xTg-AD mice led to different results. It increased Aβ loads in multiple brain regions and was accompanied with worsened cognitive functions, and E2 administration either rescued these deficits (Carroll et al., 2007, 2010b; Carroll and Pike, 2008), or caused no significant changes (Overk et al., 2012; Palm et al., 2014). Our previous study in rats has shown that the plasma E2 and brain E2 levels in the hippocampus, PFC or hypothalamus change independently following OVX (Guo et al., 2018). Therefore, the higher Aβ accumulation we observed in female 3xTg-AD mice than males would rather be related to the local brain E2 levels. Furthermore, although EGR1 and AChE levels did not vary significantly between sexes, they were both significantly negatively correlated with soluble Aβ(1–42) in pooled 3xTg-AD males but not females, which indicates that the soluble Aβ(1–42) has an inhibitory effect on the expression of EGR1 and AChE, while higher estrogens in female mice may abolish such an effect. Our findings in C99-SH-SY5Y cells support these ideas.

We first showed that increasing concentrations of Aβ(1–40) and Aβ(1–42) in the C99-SH-SY5Y cell culture medium was accompanied by decreased intracellular expression of EGR1 and AChE. This is consistent with previous findings that exposure to Aβ(1–42) led to reduced AChE expression in new neurons generated from human embryonic stem cells (Malmsten et al., 2014). In addition, intracerebroventricular Aβ(1–42) injection in rats decreased EGR1 mRNA levels in the hippocampus (Karimipour et al., 2019). These findings are also in agreement with our findings that reduced intracellular EGR1 expression may reduce AChE expression (Hu et al., 2019). Next, we observed that adding E2 to the C99-SH-SY5Y cell culture medium led to a decrease in the expression of intercellular Aβ(1–42) accompanied with increased expression of intracellular EGR1 and AChE. This is, on the one hand, consistent with previous *in vitro* studies demonstrating that raising E2 levels by adding directly to the culture medium leads to a decrease in Aβ(1–42) (Xu et al., 1998, 2006; Liang et al., 2010), on the other hand, this indicates a protective role of local E2 in the acute/early stages of AD, since our previous studies found stable or slightly increased EGR1 and AChE expression in the NBM and the PFC, which may contribute to maintaining cognitive function in early AD (Bossers et al., 2010; Zhu et al., 2016; Hu et al., 2019). Moreover, since E2 was found to activate the expression of EGR1 in some *in vitro* cancer studies (Chen et al., 2004; Vivacqua et al., 2012), the increased EGR1 after E2 supplementation could be induced directly by E2 and/or indirectly *via* reduction of extracellular Aβ(1–42).

It should be noted that we observed positive correlations between EGR1 and AChE (in mice) and the same directional changes in EGR1 and AChE expression (in cells), supporting our previous finding of EGR1 positively regulates AChE gene expression (Hu et al., 2019). However, EGR1 and AChE levels were kept stable in 3xTg-AD mice either in relation to age from 3 to 12 months, or compared with WT mice. This suggests that Aβ might not play a critical role affecting these two molecules in the hippocampal complex of this AD mouse model. Since we have previously found reduced EGR1 and AChE in the PFC of 3xTg-AD mice compared with WT mice, especially from the later age group (7–8 months) (Hu et al., 2019), the role of Aβ on the expression of EGR1 and AChE may be brain area-dependent.

We did not find positive p-Tau signal in the brains of 3xTg-AD mice until the age of 11–12 months. According to the original description of these mice, extensive p-Tau immunoreactivity appeared at about 12 months of age in the hippocampus, while Aβ developed much earlier in the 3xTg-AD mice (Oddo et al., 2003). Therefore, even though p-Tau may have eventually been expressed in our mice, as we focused on exploring the mechanism of sex differences in early AD, we studied the role of Aβ in this 3xTg-AD mouse model. Concerning the earlier appearance of Aβ in this 3xTg-AD mouse model, our findings provide some support for the amyloid cascade hypothesis which predicts that Aβ is the initiating trigger of cascade reaction of subsequent pathological changes that underlies AD (Hardy and Selkoe, 2002) and that the accumulation of p-Tau is driven by amyloid pathology (Karran and De Strooper, 2016). It should be noted, however, that since the development of p-Tau distribution is significantly correlated with cognitive decline in AD (Arriagada et al., 1992; Giannakopoulos et al., 2003), the sex differences in p-Tau in AD animal models deserves further attention in the future.

In conclusion, the clear sex difference of Aβ levels in the subiculum of 3xTg-AD mice provides a good model for studying the mechanism of the sex difference in AD. In addition, our *in vitro* findings suggested that local E2 may exert a protective effect in AD brains by activating EGR1 accompanied with reduced soluble Aβ(1–42), and thereby maintaining synaptic and cognitive functions in early AD.

## Data availability statement

The original contributions presented in this study are included in the article/Supplementary material, further inquiries can be directed to the corresponding authors.

## Ethics statement

This animal study was reviewed and approved by Animal Experimental Committee of the Royal Netherlands Academy of Arts and Sciences.

## Author contributions

Y-TH, DS, and A-MB contributed to the conception and design of the study. Y-TH, X-LC, Y-NZ, HM, JS, NB, AS, and JZ contributed to the acquisition and analysis of data. Y-TH, X-LC, DS, and A-MB drafted the text and prepared the figures. All authors contributed to the article and approved the submitted version.
