# Erratum: Sex differences in hippocampal β-amyloid accumulation in the triple-transgenic mouse model of Alzheimer's disease and the potential role of local estrogens

**DOI:** 10.3389/fnins.2023.1220955

**Published:** 2023-05-23

**Authors:** Frontiers Production Office

**Affiliations:** Frontiers Media SA, Lausanne, Switzerland

**Keywords:** Alzheimer's disease, triple-transgenic AD mouse, β-amyloid, 17β-estradiol, sex differences

Due to a production error, there was a mistake in the first sentence in the discussion paragraph of the abstract. Aβ was incorrectly given as Ap. The correct sentence reads: “This data shows that the 3xTg-AD mouse model can be useful for studying the human sex differences of AD, but only in regards to Aβ.”

The publisher apologizes for this mistake. The original article has been updated.

