# Supplementary material for: Sex differences in hippocampal β-amyloid accumulation in the triple-transgenic mouse model of Alzheimer’s disease and the potential role of local estrogens
Source: Front Neurosci. 2023 Mar 8;17:1117584. doi: 10.3389/fnins.2023.1117584 (PMC10030503; doi:10.3389/fnins.2023.1117584)
Supplement: Supplementary file 1 [file Table_1.DOCX]

**Table 1. Correlation between the mRNA level of early growth response-1 (EGR1) and acetylcholinesterase (AChE) in 3xTg-AD and WT mice**

| ***3xTg-AD mice*** | |  |  |  |  |  |  |
| --- | --- | --- | --- | --- | --- | --- | --- |
| **Entire group** | | (r=0.88, *P*<0.001) | |  |  |  |  |
| **Sex subgroup** | | **male** | | **female** | |  |  |
|  | | (r=0.93, *P*<0.001) | | (r=0.71, *P*<0.001) | |  |  |
| **Age subgroup** | | **3-4-month** | | **7-8-month** | | **11-12-month** | |
|  | | (r=0.87, *P*<0.001) | | (r=0.83, *P*<0.001) | | (r=0.91, *P*<0.001) | |
| **Sex*age subgroup** | | **3-4-month** | | **7-8-month** | | **11-12-month** | |
|  | **male** | (r=0.98, *P*=0.001) | | (r=0.88, *P*=0.009) | | (r=0.96, *P*<0.001) | |
|  | **female** | (r=0.97, *P*<0.001) | | (r=0.80, *P*=0.005) | | (r=0.64, *P*=0.06) | |

| ***WT mice*** | |  |  |  |  |  |  |
| --- | --- | --- | --- | --- | --- | --- | --- |
| **Entire group** | | (r=0.80, *P*<0.001) | |  |  |  |  |
| **Sex subgroup** | | **male** | | **female** | |  |  |
|  | | (r=0.84, *P*<0.001) | | (r=0.81, *P*<0.001) | |  |  |
| **Age subgroup** | | **3-4-month** | | **7-8-month** | | **11-12-month** | |
|  | | (r=0.82, *P*<0.001) | | (r=0.83, *P*<0.001) | | (r=0.77, *P*<0.001) | |
| **Sex*age subgroup** | | **3-4-month** | | **7-8-month** | | **11-12-month** | |
|  | **male** | (r=0.85, *P*=0.002) | | (r=0.92, *P*<0.001) | | (r=0.85, *P*=0.004) | |
|  | **female** | (r=0.80, *P*=0.009) | | (r=0.68, *P*=0.09) | | (r=0.94, *P*<0.001) | |
